# Supplementary material for: Influences on drinking choices among Indigenous and non-Indigenous pregnant women in Australia: A qualitative study
Source: PLoS One. 2020 Apr 30;15(4):e0224719. doi: 10.1371/journal.pone.0224719 (PMC7192424; doi:10.1371/journal.pone.0224719)
Supplement: Attachment — Submitted filename: Response to Reviewers.docx [file pone.0224719.s001.docx]

**PONE-D-19-29063
Pregnant women’s understanding and conceptualisations of the harms from drinking alcohol: a qualitative study**

**Authors’ response to editorial requirements (January 9 & March 3, 2020) and reviewers’ comments**

**Summary of response:**

We thank the reviewers for their comments and have taken a number of steps to improve our manuscript based on their suggestions. Primarily, we have now provided greater engagement with the issue around abstinence compliance in the context of inconsistent evidence for harm with lower levels of alcohol. We also included additional information on our sampling strategy and interview length, incorporated participant quotes within the results section and amended the manuscript title.

**Editorial requirements (January 9, 2020):**

When submitting your revision, we need you to address these additional requirements:

**1.** Please ensure that your manuscript meets PLOS ONE's style requirements, including those for file naming. The PLOS ONE style templates can be found at [http://www.plosone.org/attachments/PLOSOne_formatting_sample_main_body.pdf](https://protect-au.mimecast.com/s/GE3YCmO5OrSjzkVDUBOsDG?domain=plosone.org) and [http://www.plosone.org/attachments/PLOSOne_formatting_sample_title_authors_affiliations.pdf](https://protect-au.mimecast.com/s/DW2RCnx1xvh7vr4jHZDey8?domain=plosone.org)

***Authors’ response:*** *Thank you we have double-checked that the manuscript complies with these formatting requirements and made the necessary changes.*

**2.** We note that you have indicated that data from this study are available upon request. PLOS only allows data to be available upon request if there are legal or ethical restrictions on sharing data publicly. For information on unacceptable data access restrictions, please see [http://journals.plos.org/plosone/s/data-availability#loc-unacceptable-data-access-restrictions](https://protect-au.mimecast.com/s/nJR1CoV1VwsrE6AZiOrDIA?domain=journals.plos.org).

In your revised cover letter, please address the following prompts:

a) If there are ethical or legal restrictions on sharing a de-identified data set, please explain them in detail (e.g., data contain potentially identifying or sensitive patient information) and who has imposed them (e.g., an ethics committee). Please also provide contact information for a data access committee, ethics committee, or other institutional body to which data requests may be sent.

We will update your Data Availability statement on your behalf to reflect the information you provide.

***Authors’ response:*** *We understand that data sharing is very valuable to increase credibility and transparency of any scientific publication and there are some recent progresses to help overcome the some of the difficulties in public sharing of qualitative data. Unfortunately, the interview data collected as part of this project raises some concerns around a) the highly sensitive nature of the information provided, b) the risk of re-identifying participants as well as c) issues around consent and data ownership with special regard to d) our Indigenous Australian participants:
a) Our study participants provided information on the understanding that this would be confidential and that their identity would not be revealed. As such, they sometimes disclosed highly sensitive information about themselves or their friends, which would not be appropriate to make public, especially if taken out of context;
b) Some participants may be identified through their comments, especially those who took part at our rural and remote Indigenous health services;
c) Consent was provided with the understanding that the data would be used for the purpose of this project and public sharing of data may results in new research. While this would maximise use of the data, participants have not consented to their information being used for further research and it is their expectation that their views about alcohol use in pregnancy would only be used to inform future maternity care.
d) There are additional policies governing data involving Aboriginal and Torres Strait Islander peoples and communities, e.g. “With respect to the ownership of data and information used in or generated by research involving Aboriginal and Torres Strait Islander peoples and communities, institutions or researchers may hold data or information; however, they should not make decisions about the access to or reuse of this data or information without proper consultation with its Indigenous owners, if any.”
Taken from: Management of Data and Information in Research: A guide supporting the Australian Code for the Responsible Conduct of Research. National Health and Medical Research Council, Australian Research Council and Universities Australia. Commonwealth of Australia, Canberra (2019).*

***Suggested Data Availability Statement:*** *Data cannot be shared publicly because of confidentiality of participants. Some participants may be identified by their comments. Data are available from the Murdoch Children’s Research Institute Institutional Ethics Committee for researchers who meet the criteria for access to confidential data (contact Prof Jane Halliday at* [*janehalliday.h@mcri.edu.au*](mailto:janehalliday.h@mcri.edu.au)*).*

**Additional editorial requirements (March 3, 2020):**

**Editor:** 1. Thank you for providing the following data availability statement: "Data cannot be shared publicly because of confidentiality of participants. Some participants may be identified by their comments. Data are available from the Murdoch Children’s Research Institute Institutional Ethics Committee for researchers who meet the criteria for access to confidential data (contact Prof Jane Halliday at janehalliday.h@mcri.edu.au)."

Before we proceed, please clarify Prof. Jane Halliday's relationship to the data and their position at Murdoch Children’s Research Institute.

***Author response:*** *Certainly. Professor Halliday is Head of the Reproductive Epidemiology research group at the Murdoch Children’s Research Institute. The first, third and last authors are affiliated with Reproductive Epidemiology and the data are stored on a secure drive of this research group. Prof Halliday has high level oversight over all research conducted in her group and is named on the ethics applications listed in the ethics statement. As such, she is cognisant of the study and its data sensitivities and able to liaise with the ethics committee to facilitate data access, while not having been directly involved in the analysis and preparation of this manuscript.*

**Editor:** Please also confirm if Murdoch Children’s Research Institute Institutional Ethics Committee is the ethics committee which has imposed data sharing restrictions on the data underlying your study.

***Author response:*** *The Murdoch Children’s Research Institute Institutional Ethics Committee is the Human Research Ethics Committees of the Royal Children’s Hospital. We can confirm that for the reasons listed in our previous response, the ethics approval (#35173A&B) states that:*

*“Participants will be identifiable in the recording but code names will be applied at the point of transcription. All analyses and reporting will not identify any individual. The data will only be accessible to researchers and staff directly working on this project.”*

*We hope that we have addressed your queries about data sharing satisfactorily. If you prefer us to nominate another contact for data access, please let us know your criteria and we will attend to this ASAP.*

**Editor:** 2. Thank you for including your ethics statement on the online submission form:

"This study was carried out in strict accordance with the Australian National Statement on Ethical Conduct in Human Research (2007). Ethical approval was granted by the Australian Institute of Health and Welfare (EO2015/3/196), the Human Research Ethics Committees of the Royal Children’s Hospital (35173A&B), Cabrini Hospital (07-09-11-15), Mercy Health (R15-28), Western Health (35173A&B), Goulburn Valley Health (GVH32/15), the Research Sub-Committee of the Central Australian Aboriginal Congress Aboriginal Corporation, Alice Springs, and the Central Australian Human Research Ethics Committee (15-349).
Written consent was obtained from all study participants."

To help ensure that the wording of your manuscript is suitable for publication, would you please also add this statement at the beginning of the Methods section of your manuscript file.

***Author response:*** *Thank you, and our apologies for the misunderstanding. We have now added this statement into the beginning of the methods section as well and uploaded a new revised manuscript.*

**Reviewers' comments:**

1. Is the manuscript technically sound, and do the data support the conclusions?

Reviewer #1: Yes
Reviewer #2: Yes

2. Has the statistical analysis been performed appropriately and rigorously?

Reviewer #1: N/A
Reviewer #2: Yes

3. Have the authors made all data underlying the findings in their manuscript fully available?

Reviewer #1: No
Reviewer #2: Yes

***Authors’ response:*** *Please see our statement in point 2 of the editorial requirements on pages 1-2 of our response.*

4. Is the manuscript presented in an intelligible fashion and written in standard English?

Reviewer #1: Yes
Reviewer #2: Yes

**5.** Review Comments to the Author

**Reviewer #1:** The paper provides an interesting account of pregnant women's understanding and conceptualisation of the harms of drinking alcohol. This is an important topic. Whilst the paper has many merits, further work is required to strengthen.

***Authors’ response:*** *Thank you for acknowledging the importance of this paper and for your constructive comments and suggestions. We believe the manuscript has been strengthened as a result of the revisions.*

**Reviewer #1:** My main concern is the assumption throughout the paper that any alcohol is problematic. This is not accepted universally within the field, and there is a lack of convincing evidence for harm below 5 units of alcohol. Greater specificity is required in the intro when discussing any alcohol (including very low levels) and when the literature referenced is concerned with heavier levels.

***Authors’ response:*** *The object of our introduction was to explain that despite official pregnancy drinking guidelines recommending abstinence, many pregnant women, especially in some sub-populations, drink alcohol and therefore, this blanket abstinence message does not appear to be effective for all. The aim of the study was to understand why this is so. Our study found that one of the reasons for alcohol use in pregnancy was that some women understand there is a lack of evidence around lower level drinking and as a result they are making their own personal risk assessments. We posit informed ideas on how to change the messaging to increase adherence to guidelines.*

***Amendments to manuscript:*** *We have elaborated in the introduction on the evidence for harm to clarify this issue for the reader who may not be aware of the ongoing lack of convincing evidence for harm with lower levels of alcohol exposure and that because of this, pregnancy drinking guidelines around the world recommend that it is safest not to drink alcohol at all. There is now a new opening statement and additional citations of recent systematic reviews (lines 39-52; citation 1-3 in tracked manuscript).
In addition, we have included Table 1 to outline the current recommendations for the Australian guidelines to illustrate the framing of abstinence message within the context of the available evidence.
To assist with the flow of the new introduction, a number of small tracked changes to the text were made throughout.*

**Reviewer #1:** This issue also requires greater engagement in the discussion. In my view, it is not sufficient for the authors to cite one study (their own) to show that the women are 'wrong' when perceiving small amounts of alcohol to be low risk, given a large volume of research which has found little evidence for risk associated with lower levels (see systematic reviews: Henderson, J., R. Gray, and P. Brocklehurst, Systematic review of effects of low moderate prenatal alcohol exposure on pregnancy outcome and Gray, R., Low-to-moderate alcohol consumption during pregnancy and child development – moving beyond observational studies).

***Authors’ response:*** *We have carefully considered this comment and are convinced that our discussion does not imply that women are ‘wrong’ when perceiving small amount of alcohol to be low risk. Rather, our discussion makes the point that while there is little evidence for risk of clinically relevant harm (e.g. developmental problems in the child) with lower or sporadic alcohol exposure, it is understood that alcohol is an established teratogen altering fetal neurobiological development. Further, less clearly identifiable later mental health outcomes, such as anxiety and depression have also been implicated following prenatal alcohol exposure. In light of this and in an effort to promote adherence to abstinence guidelines, it may be helpful to reframe messages about harm to messages about optimising health outcomes for the child through abstaining (lines 449-461).*

***Amendments to manuscript:*** *We stand by our points made in the discussion as above, but have tracked some small changes to the text to articulate these better and added additional citations to lines 460-468; citations 28, 31 &32.*

**Reviewer #1:** Given the stated objectives of paper to examine influences, I was disappointed that the authors did not engage with the discrepancy that exists between a blanket no alcohol message and much of the evidence that the women are able, and choose to access. This discrepancy undermines the public health message and could have been drawn out in the findings (informing) and in the discussion.

***Authors’ response:*** *Thank you, in addition to the related improvements to the introduction and discussion detailed above, we have now also incorporated this information in the findings and discussion.*

***Amendments to the manuscript:*** *We have expanded the results by adding an extra sentence (choosing) to explain that the views of women who said alcohol consumption is an individual choice are in part based on an understanding of the lack of evidence for harm with lower levels of alcohol use (lines 312-313).
This information is also added as new sentence in the discussion (lines 393-394), an amendment to lines 423-424 and 457-458.*

**Reviewer #1:** The authors could then also explore how best to inform in light of this discrepancy in evidence/advice. Presumably if the authors believe the women need to have their misinformation corrected, proving evidence to the women of the harm is the way to go. This would be most unhelpful and may result in adverse outcomes (unplanned pregnancies where alcohol was consumed prior to knowledge of pregnancy being unduly concerned/choosing to terminate for fear of impact caused, greater mistrust of information, decreased disclosure, stigma and judgement). Whether the authors accept that any alcohol is low risk during pregnancy or not, the women appear to be influenced by the community/informal beliefs and the lack of evidence showing harm at low levels of consumption.

***Authors’ response:*** *We agree with the reviewer that women’s behaviour can be influenced by community/informal beliefs and their awareness of a lack of evidence around low levels of alcohol consumption. Abstinence messages, such as the one in Australia from where the study population was drawn, usually frame the risk of harm within the available evidence and issue practical advice for maternity care providers to assist with contextualising the abstinence recommendation. In part this is to avoid undue concern for women who may inadvertently have consumed alcohol before pregnancy recognition.*

***Amendments to the manuscript:*** *This is now articulated in more detail in the introduction and new Table1.*

**Reviewer #1:** There appears to be a mismatch between the title and the objectives. The title is focused upon the women's understanding whilst the objectives is concerned with behaviour influences. The objectives would seem much broader than the title implies.

***Authors’ response:*** *Thank you and reviewer #2 for making suggestions to improve the title to better reflect the content of the manuscript.*

***Amendments to the manuscript:*** *We have revised the title to read: Influences on drinking choices among Indigenous and non-Indigenous pregnant women in Australia: a qualitative study.*

**Reviewer #1:** Methods - further details are needed about the 'group discussions'. The language used to describe this method is not consistent and at times is referred to as a group discussion (which implies focus groups) and at others is referred to as a group interview. Why was this approach used? Some justification is given for times when it was NOT used (e.g. to increase comfort) but none for the benefits of interviewing three women together.

***Authors’ response:*** *Thank you for highlighting this inconsistency. We have amended the wording of the manuscript to be consistent. In answer to your question, we offered group discussions as a preference to encourage participants to analyse their opinions more deeply within the group dynamic. However, we were advised by staff in the Indigenous clinics that a group setting could be problematic for some women who may not feel comfortable to express their own thoughts in the presence of an elder or may experience shame discussing the topic with others from their community. The interviews done as part of this study did not warrant separate analysis as the responses did not differ from those obtained within the groups.*

***Amendments to the manuscript:*** *We replaced the term ‘group interview’ with ‘group discussion’ throughout. We also inserted a new sentence in lines 167-169 to explain why we combined the two methods of data collection in our analysis and in lines 485-488 to elaborate on why we offered both types of data collection.*

**Reviewer #1:** How were the groups formed? Was there any thought about characteristics that would make the groups appropriate/helpful to promote discussion, did the women know each other, was there group interaction (as focus group)? I understand the authors used convenience sampling. A number of factors are likely to be relevant to the women's influences. Is there any information about sample variation beyond Indigenous Australian/non Indigenous and weeks gestation?

***Authors’ response:*** *In line with our convenience sampling, the groups were formed with women attending antenatal care and who were available on the day and time of the scheduled group discussion at each setting. Convenience sampling was used because researchers travelled to each site on particular dates and able to recruit participants while in attendance. We aimed for a representative spread of health services, but did not target participant characteristics further e.g. based on particular alcohol use patterns. We did not collect demographic information on the women other than those reported in the manuscript and as such our findings must be interpreted within the context of our sample (discussion in ‘methodological considerations’).*

***Amendments to the manuscript:*** *No changes beyond those made in response to the reviewer’s previous point about our reasons for offering group discussions.*

**Reviewer #1:** Women who choose to drink a little during pregnancy, those who drink heavily and those who abstain are likely to understand and conceptualise harm from alcohol very differently. It may have been more helpful to take a purposive approach to sampling. However, as this approach was not taken, can the authors comment of levels of alcohol use within the sample? Or provide justification as to why this was not discussed.

***Authors’ response:*** *The purpose of the study was to collect women’s views on alcohol use. If women had to disclose their actual alcohol use, this would have changed the focus to participants defending their decisions. Not being required to put their views into the context of their actual drinking allowed women to speak freely and without fear of judgement. While no direct questions about personal alcohol use were asked, some women were happy to explain that they drank some alcohol, or that they abstained, and what their reasoning was.*

***Amendments to the manuscript:*** *No changes.*

**Reviewer #1:** The findings are quite thin and some of the interviews short (10 mins). This suggests a lack of depth in the data. What was the mean interview length? If this is closer to 30 mins this would provide some reassurance.

***Authors’ response:*** *The shortest individual interview (12 minutes) was with one of our Indigenous participants who was well informed about alcohol use in pregnancy and spoke very succinctly on the topics presented, needing very little prompting. Even this interview, short in duration, contributed much valuable data to inform our analysis and findings.
In relation to the depth of data, we considered that our findings are specific to the 28 women taking part and possibly their broader interactions. However, our data analysis confirmed that data saturation was reached, and no new topics arose that warranted further investigation (see discussion; methodological limitations). We believe our findings to be strong as the topics we identified cover a broad range of considerations for pregnant women around alcohol use in pregnancy, encompassing all concepts of the Health Belief Model and providing a good understanding of the women’s behaviour.*

***Amendments to the manuscript:*** *We have modified Table 2 (formerly Table 1) to show more detail on the times taken at each interview, which ranged from 12 minutes (single interview) to 39 minutes (group interview). We also added this information into the text and listed the average interview length, which was 22 minutes (median time was very similar at 23 minutes).*

**Reviewer #1:** I think the table of quotes which is presented disconnected to the analysis is unhelpful and it is unclear how the 'representative sample' has been selected. I would encourage the authors to integrate these within the findings. I am unclear what is the table and what is the figure.

***Authors’ response:*** *Thank you for this feedback, we have now incorporated the quotes within the body of the text and removed Table 2. To retain clarity of actual manuscript revisions, we have not tracked these changes, but placed the quotes in italics.*

**Reviewer #1:** **The discussion would benefit from greater depth and engagement with the literature**. In particular, as discussed above there is opportunity to engage with the mismatch between what the current approach is (aimed at influencing the women's drinking) and how the women understand harm.

***Authors’ response:*** *We trust that this issue has now been addressed in response to the reviewer’s earlier comments and suggestions in relation to guidelines recommending abstinence, women’s knowledge of the inconsistent evidence to support this recommendation with lower levels of alcohol use and their consequent personal interpretations of risk.*

**Reviewer #1:** Multiple subheadings within the discussion interrupts the discussion.

***Authors’ response:*** *We understand the reviewer’s concern about this. However, in preparing the manuscript we carefully considered the use of subheadings and believe that they are necessary to assist the reader in interpreting our findings in the context of each component of the Health Belief Model.*

**Reviewer #2:** This appears to be a very interesting and potentially useful study. The importance of the topic cannot be overstated. The use of the HBM framework to design and analyze the study is especially compelling. The presentation of the data and the Discussion sections are very thought-provoking and offer useful clinical information.

***Authors’ response:*** *Thank you for your positive comments*

**Reviewer #2:** However, I did have some questions/comments:

**Reviewer #2:** 1) The title is very general, but the sample is a very specific group of women. A title that provided a better characterization of population from which the sample is drawn might make the paper more applicable and address issues of diversity.

***Authors’ response:*** *Thank you for this suggestion, we have amended the title to better reflect the study population.*

**Reviewer #2:** 2) Along the same lines, data on how many women were available and how many actually agreed to participate would provide a better basis for seeing this very small sample as part of a larger context.

***Authors’ response:*** *As this was a convenience sample, we do not have any information on the proportion of women who agreed to take part out of the total number of women approached. Data collection included the views of 28 pregnant women on a particular topic, which in qualitative study terms is not a small sample. In our ‘methodological considerations’ section, we discussed that our sample may not be representative of the entire population, but that the data obtained did not arise any new topics which warranted further investigation, indicating that that saturation was reached. Noting, it is not the intent of qualitative studies to ever be representative in preference to generative. We believe that a larger context is provided for the reader in the discussion through the use of the Health Belief Model and connecting our findings with the literature.*

***Amendments to the manuscript:*** *No changes made.*

**Reviewer #2:** 3) The Introduction could benefit from two changes. First, there is literature on why people in general make poor drinking decisions even when they have full awareness of the potential negative consequences. Some review of this literature would be helpful. Second, the interviews appear to be informed by the Health Belief Model, but HBM is not really discussed in the introduction. I would encourage a little more of a theoretical basis for paper to appear in the Introduction, rather than waiting until the Discussion.

***Authors’ response:*** *Thank you for these suggestions. In relation to your first point, the wider literature on alcohol choices shows that higher level drinkers usually underestimate the negative effects or that the effectiveness of health warning labels is influenced by personal relevance (Winstock et al; Alcohol & Alcoholism; 2019). Alcohol choices in pregnancy appear to be influenced by application of the precautionary principle (i.e. abstinence), an awareness of the inconsistent evidence around lower level drinking, possible issues with addiction or an adverse social environment. We have included additional information on the first two in response to reviewer #1’s comments in the current manuscript revision, which we hope to be helpful here as well.
Regarding the Health Belief Model, this is not presented in the introduction/methods because we did not design the study or analyse the data based on the Health Belief Model. Our qualitative data analysis was performed inductively and not with any theoretical framework, preconception or advance knowledge in mind. The HBM framework provided a good fit to guide interpretation of our findings. Consequently HBM was introduced in the discussion section.*

***Amendments to the manuscript:*** *No changes made.*
